# Manners of English Society

**Published:** 1888-10

**Authors:** 


### MANNERS OF ENGLISH SOCIETY.

The signal deterioration of manners that has for some time been going on in what is called good society, is every year becoming more glaring and more deplorable. The delicate and subtle deference which every gentleman used to pay to every woman, because she was a woman and for no other reason whatever, is already old fashioned, and promises shortly to become obsolete. No woman now thinks of expecting from any man the polite homage which once on a time was the privilege of one sex and the honor of the other. Men come into a room full of ladies with as much indifference and *sans facon* as though they were entering the morning room of their club, and quit it with precisely the same want of ceremony and self-constraint. While in the society of women they loll, lean and almost lie at their ease, as though they were in the bosom of their own family—indeed, with a free and easy gracelessness that a generation ago no gentleman would have permitted himself among his most intimate relatives. In the first approaches of members of one sex to members of the other there is no longer any suavity, any hesitation, any well-bred reserve ; men and women who scarcely know each other act as though they were hail-fellow-well-met, had been in the playground together, and been acquainted all their lives.

The demeanor of women, now-a-days, to men, is on a par with the male behavior we have described. Far from resenting the unceremoniousness with which they are treated by men who are in reality utter strangers to them, they go to meet it half way, and permit themselves to be on a foot of familiarity—as far as manner is concerned—with the first comer, provided he seems to be one of their "own set," that could not be greater if their acquaintance had existed for years. The same "don't-care-a-hang" conduct is perceptible in the conduct of visitors and guests to their host and hostess.

The notion that people are to be specially honored in their own houses has gone quite out of fashion. No one, now-a-days, is so antiquated in his ideas as to suppose that hospitality is to be regarded as a favor conferred on the person to whom it is extended. On the contrary, it is the guest who confers an obligation by paying a call, accepting an invitation to dinner, or paying a country visit, and who has a perfect right to indulge in frank and free censure to his neighbors in case he does not find everything to his liking in the establishment he condescends to distinguish by his presence. In a word, guests, now-a-days, treat hosts and hostesses as men treat women—that is to say, as persons whom it is very good and amiable of them to notice at all. And where people really know each other intimately, the behavior of men to women, and vice versa, is such as would have appalled the least ceremonious of our fathers. Women call men by their surname, without the prefix of Mr., or even by their Christian names, abbreviated to suit the current taste for slang. And it is not shop girls or grocer's young men who do this, but ladies and gentlemen in good society.—*Anti-Ad'n Journal.*

